# Tropomodulin’s Actin-Binding Abilities Are Required to Modulate Dendrite Development

**DOI:** 10.3389/fnmol.2018.00357

**Published:** 2018-10-09

**Authors:** Kevin T. Gray, Holly Stefen, Thu N. A. Ly, Christopher J. Keller, Mert Colpan, Gary A. Wayman, Edward Pate, Thomas Fath, Alla S. Kostyukova

**Affiliations:** ^1^Voiland School of Chemical Engineering and Bioengineering, Washington State University, Pullman, WA, United States; ^2^Neurodegeneration and Repair Unit, School of Medical Sciences, University of New South Wales, Sydney, NSW, Australia; ^3^Integrative Physiology and Neuroscience, Washington State University, Pullman, WA, United States; ^4^Neuronal Culture Core Facility, University of New South Wales, Sydney, NSW, Australia; ^5^Dementia Research Centre, Department of Biomedical Sciences, Faculty of Medicine & Health Sciences, Macquarie University, Sydney, NSW, Australia

**Keywords:** neurite outgrowth, actin cytoskeleton, hippocampal neuron culture, tropomodulin, actin dynamics

## Abstract

There are many unanswered questions about the roles of the actin pointed end capping and actin nucleation by tropomodulins (Tmod) in regulating neural morphology. Previous studies indicate that Tmod1 and Tmod2 regulate morphology of the dendritic arbor and spines. Tmod3, which is expressed in the brain, had only a minor influence on morphology. Although these studies established a defined role of Tmod in regulating dendritic and synaptic morphology, the mechanisms by which Tmods exert these effects are unknown. Here, we overexpressed a series of mutated forms of Tmod1 and Tmod2 with disrupted actin-binding sites in hippocampal neurons and found that Tmod1 and Tmod2 require both of their actin-binding sites to regulate dendritic morphology and dendritic spine shape. Proximity ligation assays (PLAs) indicate that these mutations impact the interaction of Tmod1 and Tmod2 with tropomyosins Tpm3.1 and Tpm3.2. This impact on Tmod/Tpm interaction may contribute to the morphological changes observed. Finally, we use molecular dynamics simulations (MDS) to characterize the structural changes, caused by mutations in the C-terminal helix of the leucine-rich repeat (LRR) domain of Tmod1 and Tmod2 alone and when bound onto actin monomers. Our results expand our understanding of how neurons utilize the different Tmod isoforms in development.

## Introduction

Actin dynamics is crucial in both neuronal pathfinding and formation of dendrites and dendritic spines (postsynaptic component of the excitatory glutamatergic synapse; see reviews Konietzny et al., 2017; Omotade et al., 2017). In growth cones, monomeric actin (G-actin) polymerizes to form actin filaments (F-actin). Actin filaments have two characteristic ends: a fast-growing (barbed) end and a slow-growing (pointed) end. G-actin is released at the pointed end to provide a continuous supply of monomers for reincorporation at the barbed end. As monomers are added to the barbed ends, they can push on the plasma membrane and alter its shape (Doherty and Mcmahon, 2008). There are many proteins that interact with F-actin and G-actin to alter the dynamic growth and collapse of filaments (Omotade et al., 2017). Tropomodulins (Tmod) are a family of actin-binding proteins, which are known for their ability to cap the pointed end of actin filaments. Tmods’ capping ability is enhanced by binding tropomyosins (Tpms) at the pointed end (Weber et al., 1994).

Three of the four Tmod isoforms, Tmod1, Tmod2 and Tmod3, are known to be expressed in the brain (Watakabe et al., 1996; Almenar-Queralt et al., 1999; Cox and Zoghbi, 2000). Tmod1 and Tmod3 are expressed in other tissues, while Tmod2’s expression is limited to the brain (Conley et al., 2001). Tmods have two structurally distinct domains. The N-terminal domain is unstructured until binding onto the pointed end (Kostyukova et al., 2000, 2005, 2006; Greenfield et al., 2005). Tmods’ C-terminal domain has a leucine-rich repeat (LRR) domain; this domain is the second actin-binding site (Krieger et al., 2002; Rao et al., 2014). The LRR domain has a characteristic pattern of alternating β-strands and α-helices (Krieger et al., 2002); the LRR domain has a C-terminal α-helix which caps the motif and shields the hydrophobic core from the solvent.

**Figure 1:**
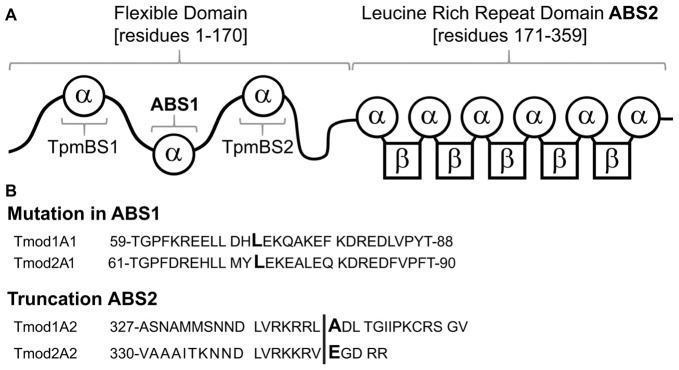
Schematic of Tmod1 and Tmod2 mutant constructs, used in this study. **(A)** A schematic of the structural and functional domains of Tmod. Residue numbering is based on Tmod1’s sequence modified from Gray et al. (2017). **(B)** Sequence alignments of Tmod1 and Tmod2 in the regions of the mutations used in this study. In the actin-binding site 1 Leu (capital/bold) was mutated to Asp. In the actin-binding site 2 the vertical line shows where the sequence was truncated by introducing a stop codon.

Tmods have four well-characterized binding sites: two actin-binding sites (ABS1 and ABS2) and two Tpm-binding sites (TpmBS1 and TpmBS2; Fowler et al., 2003; Greenfield et al., 2005; Kostyukova et al., 2005, 2006; Figure 1A). Besides capping actin filaments Tmods can use their two actin-binding sites to bind G-actin and either sequester G-actin (Fischer et al., 2006) or nucleate new actin filaments (Yamashiro et al., 2010).

There have been few studies of Tmods’ role in a neural context. Tmods’ influences on neural development have been explored both *in vivo* in knockout models and *in vitro* in cultured cell lines and primary hippocampal neurons (Cox et al., 2003; Fath et al., 2011; Moroz et al., 2013; Guillaud et al., 2014). Disruption of the *Tmod2* gene in mice resulted in several neurological deficits: reduced sensorimotor gating, hyperactivity, and impaired learning and memory (Cox et al., 2003). The knockout of *Tmod2* also caused an 8-fold increase in Tmod1 expression in the brain. Overexpression of Tmod1 or Tmod2 in cultured primary hippocampal neurons promoted dendritic complexity and increased the number of dendritic spines in an isoform-specific manner while overexpression of Tmod3 had a weak effect on dendritic arborization (Gray et al., 2016).

There are many unanswered questions remaining, regarding how Tmods regulate neuronal development and function. In this article, we address the following question: what contributions do the individual actin-binding sites of Tmod1 and Tmod2 make to these proteins’ role in neural morphogenesis? We overexpressed Tmods with mutations in the actin-binding sites in neurons and assayed morphology of dendrites and dendritic spines. We found that Tmod1 and Tmod2 require each of their actin-binding sites to specifically influence morphology. Additionally, we utilized molecular dynamics simulations (MDS), revealing Tmod isoform and mutation-dependent structural changes in Tmod structure and interactions with actin.

## Materials and Methods

### Plasmid Construction

Genes, coding wild type (WT) Tmod1 and Tmod2 were previously obtained in pCAGGs with an N-terminal Clover fluorescent protein (ClFP; Gray et al., 2016). ClFP is a derivative of green fluorescent protein (Shaner et al., 2013).

Previously our group had developed a C-terminal truncated mutant of Tmods which disrupts the actin-binding ability of the LRR domain (Colpan et al., 2016). The L71D and L73D were introduced in Tmod1 and Tmod2 respectively, using a set of two complementary oligonucleotides with codons changes for the desired mutations by *Pfu Turbo* DNA polymerase (Agilent Technologies, Santa Clara, CA, USA) with His-tagged Tmod in a pReciever-B01 plasmid as a template (Guillaud et al., 2014; Arslan et al., 2018). The original template plasmids were digested using *DpnI* (New England Biolabs, Ipswich, MA, USA), and the mutated plasmids were transformed into *E. coli* (max efficiency DH5α). All designed oligonucleotides were synthesized by Integrated DNA Technologies Inc. (Coralville, IA, USA). Subcloned sequences and introduced mutations were confirmed by DNA sequencing at GENEWIZ Inc. (South Plainfield, NJ, USA). These mutated genes were next subcloned into the pCAGGS destination vector as described previously (Gray et al., 2016) with one modification; Phusion DNA polymerase (Invitrogen, Carlsbad, CA, USA) was used in the place of place of Pfu Turbo, using the manufacturer’s protocol. For simplicity, all Tmod mutants with disrupted first and second actin-binding sites will be referred to as TmodA1 and TmodA2, respectively.

### Human Embryonic Kidney (HEK) Cell Culture

HEK cells were used as a vehicle to test that constructs expressed properly. They were transfected using Lipofectamine 2,000 using the manufacturer’s protocol (Invitrogen, Carlsbad, CA, USA). 24 h after transfection, HEK cells were lysed in RIPA buffer (Santa Cruz Biotechnology, Dallas, TX, USA). The expression of mutated Tmods was then probed for by western blotting. The primary antibodies included polyclonal rabbit antibodies against Tmod1 (custom made by Thermo Fisher Scientific, Waltham, MA, USA), Tmod2 and Tmod3 (custom made by Pacific Immunology, Ramona, CA, USA). Optimal dilutions for each antibody were determined prior to experiments. The secondary antibodies used in the experiments were Peroxidase-conjugated AffiniPure goat anti-Rabbit IgG (H + L; Jackson ImmunoResearch Laboratories Inc., West Grove, PA, USA). For each construct we saw only single bands and molecular weights corresponding to ClFP-Tmod fusion expression (data not shown).

### Protein Preparation and *in vitro* Assays

WT Tmod2 and Tmod2A1 were expressed and purified using the protocol for His-tagged Tmod2 purification described previously (Moroz et al., 2013). Relative concentrations were verified by densitometry of SDS-PAGE gels loaded with the same volumes of each protein sample. G-actin and pyrene-iodoacetamide labeled G-actin were prepared as in Ly et al. (2016). Tpm3.1 was prepared for the pointed end polymerization assay as described in Colpan et al. (2016). Actin polymerization was measured by the change in pyrene-actin fluorescence using a PTI fluorometer (Lawrenceville, NJ, USA; excitation, 366 nm, and emission, 387 nm, with 2 nm slit). One micromolar G-actin (10% pyrenyl-actin) was mixed with Tmod2 or Tmod2A1 in G-buffer (2 mM Tris-HCl pH 8.0, 0.2 mM CaCl_2_, 0.01% NaN_3_, 0.5 mM DTT, 0.2 mM ATP) and incubated for 1 min. Polymerization reactions were started by adding 20× polymerization buffer to a final concentration 25 mM Imidazole, pH 7.0, 100 mM KCl, 2 mM MgCl_2_, 1 mM EGTA at room temperature. Spontaneous actin nucleation in the absence of Tmod was measured as a control. Nucleation assays were conducted at 200 nM Tmod2 concentration. The effect on nucleation was compared by relative intensity ratios at 20 min after addition of polymerization buffer. Pointed end-capping assays were conducted as previously described (Colpan et al., 2016) with either Tmod2 or Tmod2A2 at 5 and 10 nM. Protein concentrations were measured using the difference method as described in Kostyukova et al. (2007) and Guillaud et al. (2014).

### Neuronal Cell Culture and Transfection for Morphological Studies

Hippocampi of male either female Sprague-Dawley rats (Charles River Laboratories) were harvested and dissociated on postnatal day 0–1 (P1-P2). No statistically significant differences were observed between sexes. The dissociated cells were then plated at a density of 3.4 × 10^4^ cells per cm^2^ on glass coverslips precoated with poly-L-lysine (Sigma; molecular weight 30,000) in 24-well plates with 1.9 cm^2^ bottom surface area per well. Cells were kept in Neurobasal A medium (Invitrogen) supplemented with B27 (Invitrogen) as described previously (Brewer, 1997). Cultures were fixed with 4% paraformaldehyde in 60 mM PIPES, 25 mM HEPES, 5 mM EGTA, 1 mM MgCl_2_, and 87.6 mM sucrose at room temperature for 20 min. Fixed neurons were washed with PBS. Transfected and fixed neurons were permeabilized using 0.1% Triton X-100 detergent (Bio-Rad Laboratories) in PBS for 20 min before washing with PBS. Cover slips were then mounted onto microscope slides using Elvanol. Cells used for morphological experiments were transfected with Lipofectamine 2,000 (Invitrogen) using manufacturer’s protocol. Dissociated Hippocampal neurons were transfected on the 7th day *in vitro* (DIV7) for all dendrite and dendritic spine experiments. Neurons were fixed on DIV9 for dendrite experiments and DIV12 for dendritic spine experiments.

### Fluorescent Microscopy and Image Quantification

Fluorescent images for dendrite and dendritic spine experiments were taken using an Olympus IX81 inverted confocal microscope (Olympus Optical) with a 60× oil-immersion lens, numerical aperture 1.4 and resolution 0.280 μm using Slidebook 5.5 Digital Microscopy Software. Dendritic spine z-stack images were processed using MetaMorph software from Molecular Devices (Sunnyvale, CA, USA). A minimum of 29 neurons were traced for each experiment. A minimum of 20 dendritic fragments were counted for dendritic spine experiments. Neurons within each experimental condition were selected with similar levels of fluorescence corresponding to Tmod overexpression. Dendrites and dendritic spines were traced using the NeuronJ plugin (Meijering et al., 2004; Meijering, 2010; Schneider et al., 2012). Spines were counted and sorted manually using previously described criteria (Harris et al., 1992; Lesiak et al., 2014).

### Proximity Ligation Assay

Primary mouse hippocampal neurons were plated at a density of 70 × 10^3^ cells on 12 mm glass coverslips (Menzel) as previously described (Fath et al., 2009). Forty-eight hours after plating, cells were transfected with the plasmids, coding Tmod1, Tmod1A1, Tmod1A2, Tmod2, Tmod2A1 or Tmod2A2, using Lipofectamine 3000. Cells were fixed 24 h post transfection with 4% PFA in PBS for 15 min and then permeabilized with 0.1% Triton X-100 in PBS for 5 ms. The assay was carried out using the Duolink kit (Sigma Aldrich) in accordance to the manufacturer’s protocol. The primary antibodies used were anti-Tpm3.1/2 (clone 2G10.2) and rabbit polyclonal anti-GFP (1:750; Abcam) and anti-β3-tubulin (pan-neuronal marker; Millipore no. ab9354). For PLA analysis, PLA fluorescence images were thresholded and PLA puncta counted with the “Analyze Particles” function, using Fiji/ImageJ (1.51s) software. PLA puncta were counted in the cell bodies, neurite shafts and neurite tips. Since axonal and dendritic differentiation is not yet clearly identifiable at 3 DIV, using compartment-specific markers, such as Tau-1 and MAP2, we analyzed particle counts in neurites, excluding the longest neurite of each cell. This was done in order to correlate the proximity data in nascent neurites that are likely to differentiate into dendrites with the dendritic phenotype reported in this study. We chose 3 DIV for this analysis because at later stages, the growing tips of the neurites are too small in size and do not allow the same spatial segregation of puncta. Puncta in the neurites were counted in the 50 most proximal microns of the neurites and in the neurite tips, which were defined as the five most distal microns of the neurites analyzed. No PLA signal was detected in controls in which only 2G10.2 or only anti-GFP antibodies were used. For statistical analysis, 9–15 transfected cells, taken from two independent experiments, were analyzed for each experimental group (Tmod1, *n* = 10; Tmod1A1, *n* = 15; Tmod1A2, *n* = 14; Tmod2, *n* = 9, Tmod2A1, *n* = 15; Tmod2A2, *n* = 15). We tested for any morphological changes in the soma and neurite tip area as a result of destroying ABS1 or ABS2. Soma and tip area were measured, using ImageJ software.

### Molecular Dynamics Simulations (MDS) of the LRR Domains of Tmod2s and Truncated Mutants Alone and Bound on G-Actin

X-ray and simulated structures were visualized using UCSF CHIMERA (Pettersen et al., 2004). A structure of Tmod1’s LRR domain bound onto gelsolin was published (PDB code 4PKI; Rao et al., 2014). The amino-acid sequence of Tmod2 has no insertions or deletions relative to Tmod1 in the LRR domain. Over the length of the fragment in LRR domain in 4PKI, the sequence identity between 4PKI and mouse Tmod2 is 61% identity and 36% similarity. The high level of similarity between the LRR domains led us to believe that the LRR domain from 4PKI can be used to predict the LRR domains of Tmod2. Preliminary coordinates for residues of Tmod2 LRR domain were obtained by editing the PDB file of Tmod1 with the residues from Tmod2. Residues were changed to the target LRR domain through the built in function swappaa function of UCSF Chimera (Pettersen et al., 2004). For the truncated mutants, the final residues were deleted from PDB files using the del command in UCSF chimera (Pettersen et al., 2004).

MDS were run using the AMBER11 suite of codes. Hydrogen atoms were added to the X-ray structure. Counter ions (Na^+^ or Cl^−^) were added using a Coulombic potential on a grid to insure charge neutrality. The protein was then placed in a box of TIP3P water molecules (Jorgensen et al., 1983) with a minimum 10 Å distance from the protein to the edge of the box of waters. Periodic boundary conditions were used to insure the maintenance of the ensemble and statistical mechanical measures. The protein-water system was then energy minimized using 1,500 steps of steepest descent and 1,500 steps of conjugant gradient minimization. The time-evolution of the system was followed using the particle mesh Ewald method (Darden et al., 1993; Essmann et al., 1995; Hawkins et al., 1996) for calculating the electrostatic part of the potential-energy term at constant pressure, with gradual heating to physiological temperature. Temperature was maintained via coupling to an external bath using the Berendsen algorithm (Berendsen et al., 1984). The SHAKE algorithm was employed (Ryckaert et al., 1977) with a 2 fs time step. Simulations were run for 40 ns and then restarted for an additional 40 ns with the Amber function iwrap active. Root-mean-square deviation (RMSD) calculations were performed over residues 5–160’s α-carbons using the PTRAJ software (Roe and Cheatham, 2013). Surface plots were generated in UCSF Chimera using the surface function (Sanner et al., 1996) and colored using the Kyte-Doolittle scale for hydrophobicity (Kyte and Doolittle, 1982). The angles between α-helices were determined axes/planes/centroids feature in UCSF Chimera. The surface area of exposed residues was retrieved from UCSF Chimera’s attributes calculations and used to calculate changes in surface area. RMSD calculations were performed to verify that the simulations reached a new pseudo-steady state.

After the structures of the free LRR Domains were developed, they were then mapped onto 4PKI using the first 42 residues’ α-carbons of the LRR Domain. Tmod1/actin structure (PDB #4PKI) includes the methylated histidine frequently observed in actin at position 73. Since this post-translational modification is necessary for ATP hydrolysis (Nyman et al., 2002), a force field was developed for this residue and included in the simulations. Gaussian was used to assign charges on the side chain and converted into a prep file using antechamber. MDS were set up as before but run for 8 ns. A simulation of free actin was run as a control to verify that any structure changes observed in actin are caused by binding Tmod and are not an artifact of the simulation itself. This simulation was prepared by removal of all residues from Rao et al.’s (2014) structure that were not associated with actin.

### Statistical Analysis

Data are presented as mean values ± standard error of the mean (SEM). For neurite outgrowth experiments, Unpaired *t*-test for normally distributed samples or Mann-Whitney test for non-normally distributed data was used to test for statistical significance; *P* < 0.05 is considered significant. For spine morphology analyses, one-way ANOVA, followed by Tukey’s multiple comparisons test was used to determine statistical significance; *P* < 0.05 is considered significant. For PLA assays, puncta count in cell bodies, neurite shafts and neurite tips are presented as box blots and were analyzed for normality of distribution. Data showed normal distribution of puncta in cell bodies but non-normal distribution in neurite shafts and tips. Therefore, the data were tested for significant changes using one-way ANOVA, followed by Tukey’s multiple comparison (for puncta in cell bodies) or Kruskal-Wallis one-way ANOVA, followed by Dunn’s multiple comparison (for puncta in neurite shafts and tips); *P* < 0.05 is considered significant.

## Results

### L73D Mutation Affects Nucleation but Not Actin Capping Abilities of Tmod2

Our first goal of this work was to explore the role of Tmods’ actin-binding ability in neuronal development. Previously, the mutation L71D and removal of 15 residues from the C-terminus were shown to disrupt Tmod1’s first and second actin-binding sites, respectively (Kostyukova and Hitchcock-DeGregori, 2004; Kostyukova et al., 2005). Homologous mutations in Tmod2 (Figures 1A,B) disrupted its ability to bind G-actin in the corresponding actin-binding site (Arslan et al., 2018); the truncation was shown to affect Tmod2’s actin nucleation ability (Colpan et al., 2016). Here, we use four mutated Tmods, Tmod1(L71D), Tmod1(1–344), Tmod2(L73D) and Tmod2(1–346); we refer to these mutated proteins as Tmod1A1, Tmod1A2, Tmod2A1 and Tmod2A2 respectively. We expected that disruption of Tmod2’s first actin-binding site will affect its actin-nucleation ability due to inability of Tmod2A1 to bind two G-actin molecules. Recombinant Tmod2 and Tmod2A1 were expressed in *E. coli* and purified. We compared nucleation abilities of Tmod2A1 and WT Tmod2 using pyrene actin polymerization assay; the intensity of the fluorescence is proportional to the amount of F-actin. As expected, the L73D mutation abolished Tmod2’s nucleation ability (Supplementary Figure S1). This result is consistent with our previous finding that destroying the actin binding site 2 disrupts the nucleation ability of Tmod2 (Colpan et al., 2016; Arslan et al., 2018). We conclude that Tmod2 requires both actin-binding sites, ABS1 and ABS2, to nucleate filaments.

We then explored the impact of disrupting the actin-binding site 1 on Tmod2’s pointed end capping ability. Earlier we showed that disruption of the second actin-binding site of Tmod2 did not appreciably reduce its ability to cap Tpm3.1-decorated filaments (Colpan et al., 2016). We found that Tmod2A1 had the same capping ability as Tmod2 (Supplementary Figures S2A,B). This indicates that as long as either actin-binding site is functional, Tmod2 maintains the majority of its capping ability.

### Tmods Require Both Actin-Binding Sites to Modulate the Dendritic Arbor

**Figure 2:**
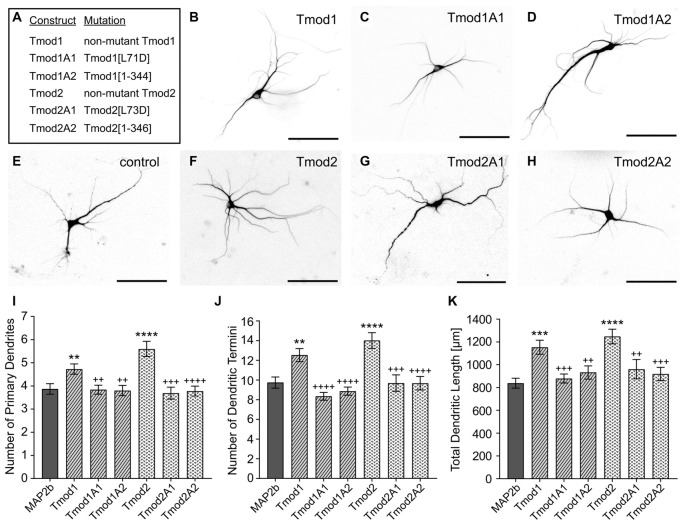
Tmod1 and Tmod2 require their actin-binding sites to modulate dendritic morphology. Primary hippocampal neurons were co-transfected with a pCAGGs plasmid encoding ClFP-tagged Tmod, wild type (WT) or with disrupted actin-binding sites (constructs shown in **(A)**), and a plasmid encoding RFP-MAP2b which was used as a dendritic marker. **(B–H)** Representative images of neurons at 9 days *in vitro* (DIV), analyzed in this experiment. Shown images are RFP fluorescence signals (scale bars = 100 μm). **(I–K)** Imaged neurons were analyzed for number of primary dendrites (left), dendritic termini (center) and total dendritic length (right). 29–55 neurons were analyzed for each condition, data pooled from two cultures with no statistical differences between controls. Error bars indicate standard error of the mean (SEM). Asterisks and plus symbols indicate statistically significant difference from the control and overexpression of WT Tmods, respectively (Unpaired *t*-test for normally distributed samples, Mann Whitney test for non-normally distributed samples, **P < 0.01, ***P < 0.001, ****P < 0.0001, ^++^*P* < 0.01, ^+++^*P* < 0.001, ^++++^*P* < 0.0001).

Previously, we showed that Tmod1 and Tmod2 overexpression alters the development of the dendritic arbor (Gray et al., 2016). We tested if Tmod1 and Tmod2 require both actin-binding sites to influence dendritic morphology. Primary hippocampal neurons were transfected with constructs to express Tmods, WT or with the disrupted actin-binding sites (Figure 2). We quantified the number of primary dendrites, dendritic termini, and total dendritic length. Tmod1 overexpressing neurons had 22 ± 6%, 29 ± 7% and 31 ± 7% more primary dendrites, dendritic termini and total dendritic length, respectively than control neurons. Neurons overexpressing Tmod2 had 45 ± 6%, 44 ± 8% and 42 ± 6% more primary dendrites, dendritic termini and total dendritic length, respectively, as compared to control neurons. All neurons expressing mutated Tmods were similar to the control; they had significantly fewer primary dendrites, fewer dendritic termini and less total dendritic length than neurons overexpressing corresponding WT Tmods (Figure 2B, *P* < 0.05). No impact on cell soma size was observed in response to Tmod1 or Tmod2 overexpression (Supplementary Figure S3). Based on these data, we conclude that Tmod1 and Tmod2 require both of their actin-binding sites to regulate dendritic morphology.

### Tmods Utilize Both Actin-Binding Sites to Specifically Alter Dendritic Spine Morphology

**Figure 3:**
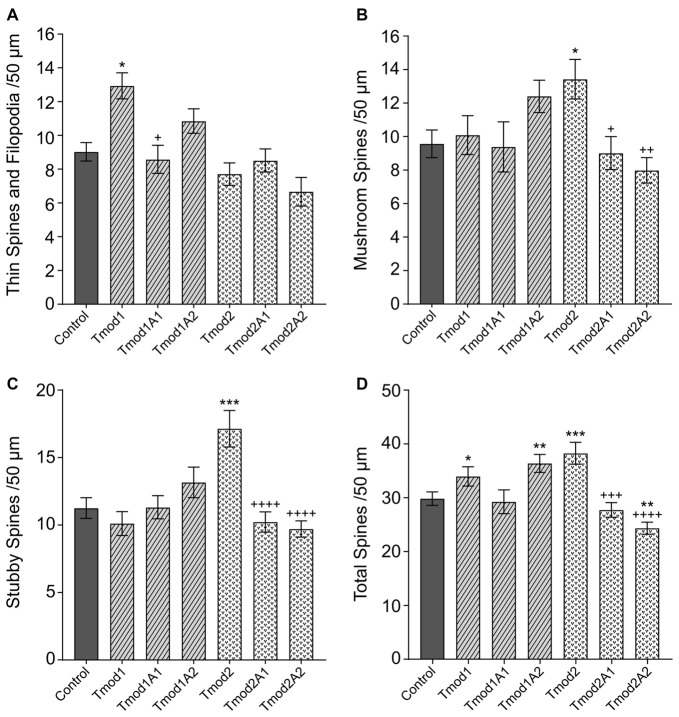
Tmod1 and Tmod2 utilize their actin-binding sites to modulate dendritic spine morphology. Primary hippocampal neurons were transfected with pCAGGs plasmid encoding ClFP-tagged Tmod, WT or with disrupted actin-binding sites. RFP-Actin was co-overexpressed to quantify dendritic spines. Neurons were analyzed at 12 DIV. 25–34 dendritic fragments were analyzed per condition. Error bars indicate SEM. Asterisks and plus symbols indicate statistically significant differences from controls and WT overexpression, respectively (one-way ANOVA, Tukey’s multiple comparison test **(A–C)** and unpaired *t*-test **(D)**, *P < 0.05, **P < 0.01, ***P < 0.001, ^+^*P* < 0.05, ^++^*P* < 0.01, ^+++^*P* < 0.001, ^++++^*P* < 0.0001).

In addition to altering the dendritic arbor, Tmods also have isoform-specific impacts on the morphology of dendritic spines (Gray et al., 2016). Overexpression of Tmod1 or Tmod2 caused the increase of thin spines or mushroom/stubby spines, respectively. We tested if Tmod1 and Tmod2 require their actin-binding sites to influence the morphology of dendritic spines (Supplementary Figure S4A). We quantified the number of filopodia/thin spines, mushroom spines and stubby spines (see classifications in Supplementary Figures S4B,C) in primary hippocampal neurons overexpressing Tmods, WT or with disrupted actin-binding sites (Figure 3). For Tmod2, the overexpression of either mutant failed to increase the number of mushroom or stubby spines. We found that the mutations diminished the effect of Tmod2 overexpression on spine morphology. Overexpression of Tmod1 mutants, Tmod1A1 and Tmod1A2 did not significantly affect the number of thin spines, compared to control. Overexpression of Tmod1 and Tmod2 led to an overall increase in spine numbers compared to control (Figure 3D), consistent with our previous study (Gray et al., 2016). Disruption of ABS1 but not ABS2 of Tmod1 eliminated the Tmod overexpression-dependent increase in total spine numbers. Disruption of both ABS1 and 2 of Tmod2 eliminated the Tmod2 overexpression-dependent increase in total spine formation.

### Tmod’s Actin-Binding Sites Are Required for Co-localization With Tpm3.1 and Tpm3.2 (Tpm3.1/2) in Primary Hippocampal Neurons

**Figure 4:**
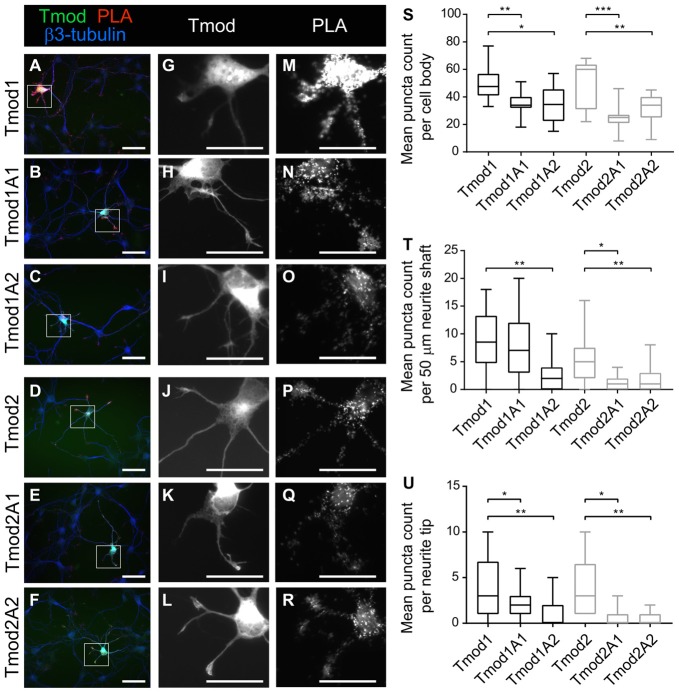
Disruption of Tmod1’s and Tmod2’s actin-binding sites weakens their association with Tpm3.1/2 *in vivo*. Proximity Ligation Assay (PLA) was used to test for close proximity of exogenous ClFP-tagged Tmods and endogenous Tpm3.1/2. Hippocampal neurons, expressing WT Tmod1 **(A,G,M)**, mutated Tmod1 **(B,C,H,I,N,O)**, Tmod2 **(D,J,P)** or mutated Tmod2 **(E,F,K,L,Q,R)** were fixed at 3 DIV and examined by PLA. Puncta of PLA reaction were counted in cell bodies, neurite shafts and neurite tips as show in graphs **(S,T,U)**, respectively. 9–15 neurons were analyzed per condition. Scale bars = 50 μm **(A–F)** and 40 μm **(G–R)**. Statistical analysis: **(S)** unpaired *t*-test for normally distributed samples **(S,T,U)** and Mann-Whitney for non-normally distributed samples **(T,U)**. *P < 0.05. **P < 0.01, ***P < 0.001.

*In vitro* assays of Tmods’ capping ability have indicated that Tmods’ actin-binding ability in the individual site is largely dispensable for the capping ability when Tpm is present (Colpan et al., 2016). However, each actin-binding site is necessary for their actin-nucleation ability of Tmod (Supplementary Figure S1, Colpan et al., 2016). We conducted proximity ligation assays (PLAs) using exogenous WT and mutated Tmods with endogenous Tpm3.1/2 to test if disruption of Tmods’ actin-binding sites reduces Tmods’ ability to associate with the pointed ends of actin in neurons (Figure 4). We assume that Tpm3.1/2 is predominantly associated with actin filaments, which leads us to believe that this assay indicates Tmods’ association with actin pointed ends. Both Tmod1 and Tmod2 produced PLA signals as shown previously (Gray et al., 2016). This indicates that the exogenous Tmod and Tpm3.1/2 are within the theoretical maximum detection distance of the assay, 30–40 nm; though, it should be noted that the functional distance is similar to that of FRET techniques (Weibrecht et al., 2010). Interestingly, neurons transfected to express the mutated Tmods with disrupted actin-binding site produced PLA signals at significantly reduced levels, compared to WT Tmods at developmental stages, preceding the time point at which changes in dendritic morphology were analyzed. Reduction in PLA puncta of mutant Tmods was observed across different regions of the neuron. Specifically, a significant attenuation was observed within the cell body, neurite shafts and neurite tips of all mutants, with the exception of Tmod1A1 in the neurite shaft region (Figure 4). Our analysis further shows that the reduction of puncta counts is not confounded by an effect of the disruption of ABS1 or ABS2 on soma area or neurite tip area (Supplementary Figure S5).

### Structure Simulations of Tmods’ LRR Domains

We sought to characterize the effects of the truncations on the structure of the LRR domains of Tmod1 and Tmod2 and their interaction with actin using MDS approach. The structure of Tmod1’s LRR domain bound onto actin (PDB 4PKI) was used as an initial structure (Rao et al., 2014). To prepare a structure of Tmod2’s LRR domain for simulations, first, we removed all of the actin residues and replaced the residues of the Tmod1’s structure with the corresponding residues of Tmod2’s LRR domain. The last 10 C-terminal residues of the Tmod1’s LRR domain were not resolved in Rao et al.’s (2014) structure and there are no analogous residues in Tmod2 (Figure 1). To prepare the truncated structures for simulations, we removed the five C-terminal residues from both Tmod1 and Tmod2.

**Figure 5:**
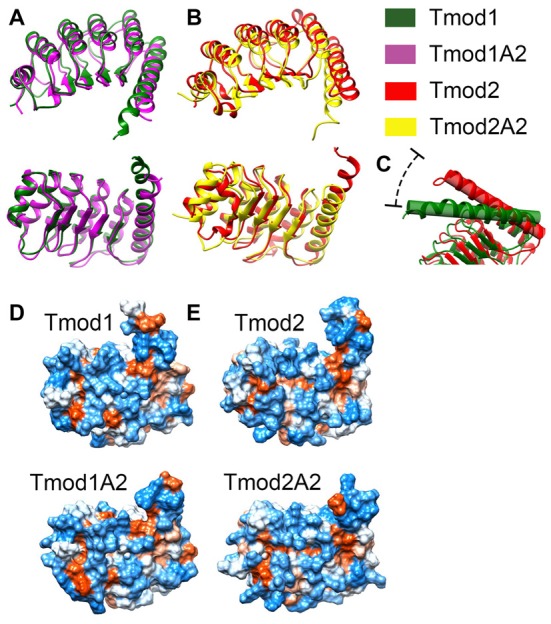
Simulated structures of the leucine-rich repeat (LRR) domains of Tmod1, Tmod1A2, Tmod2 and Tmod2A2. Representative images from the final frame of simulations for Tmod1 (green) and Tmod1A2 (purple) **(A)**. Representative images from the final frame of simulations for Tmod2 (red) and Tmod2A2 (yellow) **(B)**. The angle between C-terminal the C-terminal α-helices. Shown are the simulations for Tmod1 (green) and Tmod2 (red) **(C)**. Structures are matched over the first 35 residues’ α-carbons. Hydrophobic surface plots of representative structures by Kyte-Doolittle scale with increasing hydrophobicity from blue to white to red. Structures are viewed from the convex, β-sheet containing side, of the LRR domain are shown for Tmod1 andTmod1A2 **(D)**; Tmod2 and Tmod2A2 **(E)**.

Representative images from the final frame of each simulation are shown in Figures 5A,C. The truncation of Tmod1 caused only modest structural differences in our simulation. The Tmod2 structures had considerable differences compared to the Tmod1 simulations (Figure 5B). In the WT Tmod2 simulation, short α-helices formed starting at either end of the first β-strand in the LRR domain, which were not observed in the simulated structure of the truncated domain.

The C-terminal α-helix is of particular interest as we previously showed Tmod2’s C-terminal α-helix is more flexible than Tmod1’s C-terminal α-helix (Guillaud et al., 2014). We noticed an angling of the C-terminal α-helix in the simulated structures of Tmod2 and Tmod2A2, relative to Tmod1. There is a 24.5° angle between the C-terminal α-helices in Tmod1 and Tmod2 simulated structures (Figure 5C). The four C-terminal residues of the WT Tmod2’s simulated structure (G348-R351) are no longer constrained to an α-helical pattern seen in the Tmod1 simulated structure.

There is a 7.7° and 4.1° angle between Tmod1 and Tmod2’s truncated and WTs simulated structures, respectively. The truncation of Tmod1’s LRR domain did not seem to cause any other significant structural differences. In contrast, the truncation of Tmod2 seems to have further weakened the C-terminal end’s structure allowing three more C-terminal residues (R345-E347) to escape the α-helical structure.

Hydrophobic surfaces were developed for each of the simulations using the Kyte-Doolittle scale (Figures 5D,E). We found that there are apparent changes in the hydrophobicity of the β-sheet surface that interacts with actin. There is a hydrophobic pocket local to the αβ-loop closest to the C-terminal α-helix. This pocket is obscured in the Tmod2A2 simulated structure by the slight movement of the C-terminal α-helix. A closer examination of the exposed surface area of the residues involved in this pocket (numbered relative to the N-terminus of Tmod2, 154–155, 157, 170–171 and 174) have a modestly altered exposed surface area. In Tmod2 these residues have a total exposed surface area of 414.6 Å^2^. The C-terminal truncation of Tmod2 causes a reduction of 5.1 Å^2^. The exposed surface area was farther categorized as either solvent accessible or solvent excluded surfaces, SAS and SES respectively. Tmod2 has an SAS of 230.7 Å^2^ and SES of 183.8 Å^2^. The truncation of Tmod2 causes an SAS reduction of 8.4 Å^2^, a −4.5% change. Conversely, the SES increases by 3.3 Å^2^, a +1.4% change. These results indicate that much of the variation observed is from a rearrangement of these surfaces in this pocket as opposed to loss of the total surface area.

**Figure 6:**
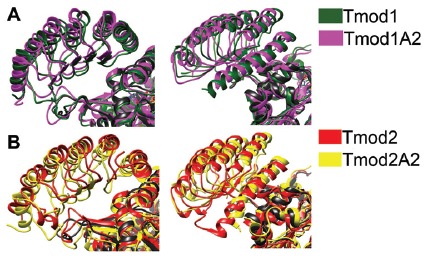
Simulated structure of the LRR domains of Tmod2 and truncated mutants binding onto actin. The resulting structures from the previous simulations were matched onto the Tmod1/actin structure (PDB #4PKI) and simulated for 8 ns. **(A)** Representative images from the final frame of simulations for Tmod1 (green) and Tmod1A2 (purple); **(B)** Tmod2 (red) and Tmod2A2 (yellow). All structures include the actin control simulation for the reference (black). Structures are matched over the α-carbons of actin in each simulation.

We next wanted to test for the impact that the simulated structural differences had on the Tmod-actin complex. The resulting structures of the LRR domains were matched over the α-carbons of the first 42 residues of the LRR domain of the Tmod1/actin structure (PDB #4PKI). The original LRR domain in the PDB structure was then deleted and simulations were run for 8 ns (Figure 6).

There were modest structural differences between the Tmod1 and Tmod1A2 simulated structures, the angle between the final α-helices was decreased by 3° (Figure 6A). In contrast, there are considerable shifts in the final α-helices of Tmod2 caused by the truncation; the angle between Tmod2 and Tmod2A2’s simulated structure increased by 5.6° and the rotational angle along the helical axis increased by 5.4° (Figure 6B). Furthermore, the α-helix of the Tmod2A2 simulation has shifted along the helical axis towards the βα-loops.

## Discussion

There are still many questions about Tmods in the nervous system that need to be explored. Tropomodulin family of proteins is known to influence a wide range of cell types, including red blood cells, muscle cells and neuronal cells (Cox et al., 2003; Ono et al., 2005; Sui et al., 2014). Although these proteins have a wide impact, our understanding of them in neural development is startlingly narrow.

Tmod1 and Tmod2 had diffuse localizations in neurons. However, Tmod1 tends to localize to leading edges in developing neurites while Tmod2 tends to be farther from the cellular membrane (Fath et al., 2011). Previous studies have shown in neural cells that Tmod2 and a Tmod2-regulating miRNA are necessary for synaptic remodeling (Hu et al., 2014), and that Tmod2 modulates long-term potentiation (LTP; Cox et al., 2003). This may happen through Tmods’ isoform-dependent effects on the dendritic arbor and dendritic spines (Gray et al., 2016). Tmod1 promotes dendritic complexity more distal from the soma, whilst Tmod2 promotes complexity more proximally. Tmod1 promotes formation of immature dendritic spines while Tmod2 promotes formation of mature dendritic spines (Gray et al., 2016).

In our previous work, we have shown that Tmod1 and Tmod2 have a differential reliance on their Tpm-binding sites to modulate neural morphology (Gray et al., 2016). Disruption of Tmod1/ tropomyosin binding abolishes the overexpression phenotype of Tmod1, whilst we observed no difference in morphology after disrupting Tmod2/tropomyosin binding. The goal of this study was to understand the roles of Tmods’ actin-binding sites in neuronal development, specifically in the dendritic arbor. Here, we found that both Tmod1 and Tmod2 utilize their actin-binding sites to modulate neural morphology and their localization in proximity to Tpm3.1/2. The mutations in each site that disrupt binding of Tmod2 to actin abolished Tmod2’s actin nucleating ability but had no impact on its capping ability of Tpm3.1-decorated filaments (Supplementary Figures S1, S2; Colpan et al., 2016; Arslan et al., 2018). Tmods require both actin-binding sites for their full actin nucleation ability, Tmod2 being essentially better nucleator than Tmod1 (Yamashiro et al., 2010; Colpan et al., 2016). Disrupting the actin-binding sites in Tmod1 and Tmod2 ameliorates the overexpression phenotype on neuronal morphology, suggesting that these sites are integral to this function (Figure 2).

Actin-binding site 1 in Tmod1 appears to be more critical for thin spine formation than actin-binding site 2, as disruption of this site significantly reduces the Tmod1 overexpression-dependent increase in thin spine formation (Figure 3A). This difference in the effect of disrupting ABS1 vs. ABS2 of Tmod1 may also contribute to the difference in promoting the total number of spines by Tmod1 overexpression (Figure 3D). Notably though, also disruption of actin-binding site 2 eliminates the significant increase in Tmod1 overexpression-dependent thin spine formation as compared to control (Figure 3A). Tmod2 requires both actin-binding sites 1 and 2 to cause the overexpression phenotype in stubby and mushroom spine formation. This difference in the impact of the mutations may be explained if Tmod2’s nucleation ability is more important for stubby and mushroom spine formation than its pointed end-capping ability.

The ability of Tmod2 to promote the formation of mature dendritic spines, a process often attributed to LTP, is in part contradictory to previous research. Hu et al. (2014) found Tmod2 to be involved in the shrinkage and elimination of dendritic spines during long term depression (LTD) induction (Hu et al., 2014), and Cox et al. (2003) observed enhanced LTP in mice, lacking Tmod2 (Cox et al., 2003). However, the study by Hu et al. (2014) does not morphologically distinguish between the types of eliminated spines. The study by Cox et al. (2003) reports a massive compensatory increase in the expression of Tmod1 in response to the elimination of Tmod2. It could be speculated that a compensatory increase in Tmod1 may lead to an increase in thin spines. These are then able to transform into mature spines in response to LTP induction, resulting in enhanced LTP. Induction of LTP is suggested to involve a two-phase process: (1) a transient increase in spine head volume, followed by (2) a longer lasting small increase in spine head volume in smaller spines along with changes in spine neck widths (Chazeau and Giannone, 2016; Hlushchenko et al., 2016). This re-organization of the cytoskeleton requires the recruitment of a plethora of actin-associating proteins. The involved processes are highly complex and work synergistically. The increase in stubby and mushroom spines, observed in response to Tmod2 overexpression, may contribute to the initial transient increase in spine volume. However, this is not integral for long-lasting, stable LTP. We postulate that Tmod2 may have a complex role within dendritic spines, which is reliant on its actin-binding sites and is potentially involved in both LTD and LTP processes. Electrophysiological experiments, incorporating Tmod mutants, are required to elucidate a functional role of Tmod in synaptic transmission further.

Delving deeper into the molecular scale, the structural differences between Tmods’ LRR domains are poorly understood. Previously, we gathered data that indicate that Tmod2’s LRR domain is less stable than Tmod1’s and localized structural differences to the C-terminal α-helix (Guillaud et al., 2014). The most-complete structure of Tmod1’s LRR domain was resolved while bound on actin (Rao et al., 2014). A large question that rises from this structure is the role of Tmods’ last C-terminal residues in binding with actin. Based on the structure, Tmod1’s residues A345-V359 as well as Tmod2’s residues E348-R352 do not seem to interact with actin; however, they are apparently necessary for actin binding (Colpan et al., 2016). How do we reconcile these two disparate pieces of information? Although our simulated structures indicate small changes in the structure of the C-terminal helix of Tmod1 and Tmod2 caused by the truncation, they do not seem to be sufficient to explain a total loss of binding in the actin-binding site 2. One possible explanation is that these C-terminal residues are important for a transient initial interaction with G-actin which then allows for the situation of the rest of the domain onto actin. Rao et al.’s (2014) structure was solved using a Tmod1-gelsolin fusion, in this case, gelsolin may have served a similar function which allowed for crystallization of the complex.

It appears that Tmods’ actin-binding sites are important components in Tmods’ ability to regulate neuronal morphogenesis. The effects on neuronal morphology are potentially partly due to the disruption of Tmod/pointed-end actin filament capping throughout the cell, including within the cell body, neurite shafts and neurite tips (Figure 4).

Taken together, these results imply that Tmod1 and Tmod2 have distinct roles in neuronal morphology that are reliant on specific sites within the protein structure. Tmod1 appears to be more important for pointed end-capping, driving immature spine formation through elongated actin filaments. This ability seems to be more reliant on Tpm-binding sites than actin-binding sites. However, Tmod2, appears to be more important for actin nucleation, which is dependent on the activity of Tmod/actin binding sites. Both Tmod1 and Tmod2 require both actin-binding sites to regulate neuronal morphology. This study and others previously utilized Tpm3.1 to examine Tpm/Tmod binding and pointed end-capping (Gray et al., 2016). However, a recent study found tropomyosin Tpm4.2 to be the major post-synaptic Tpm isoform (Suchowerska et al., 2017). It will be important to investigate the interactions between Tmods and Tpm4.2 to gain a better understanding of Tmod activity at post-synaptic sites and in neuronal morphogenesis and function.

## Ethics Statement

All procedures were conducted in accordance with the Australian Code of Practice for the Care and Use of Animals for Scientific Purposes or the Guide for the Care and Use of Laboratory Animals (Department of Health and Human Services) and were approved by the University of New South Wales Animal Care and Ethics Committee or by the Washington State University Animal Care and Use Committee, respectively.

## Author Contributions

AK, TF and GW supervised the project. AK, TF, KG and GW designed the research. KG and HS performed the research and analyzed the data. TL, CK and MC prepared proteins and conducted actin polymerization experiments. KG, AK, HS and TF wrote and revised the article. All authors read and approved the final manuscript.

## Conflict of Interest Statement

The authors declare that the research was conducted in the absence of any commercial or financial relationships that could be construed as a potential conflict of interest.

## Supplementary Material

FIGURE S1 The L73D mutation abolishes Tmod2’s nucleation ability. **(A)** Actin nucleation by 200 nM Tmod2 or Tmod2A1, control is actin alone. **(B)** Degree of actin polymerization calculated as a percentage of corresponding fluorescence in the control. Averages and standard deviations (*n* = 4–5) are demonstrated by columns and error bars, respectively. Asterisks indicate statistically significant differences between groups (****p* < 0.001) using one-way ANOVA with Holm-Sidak *post hoc* test.

FIGURE S2 Capping activity of Tmod2 and mutant Tmod2A1. **(A)** Pyrene actin pointed end-capping assay. **(B)** Comparison of fluorescence measurements after 40 min for pyrene actin pointed end capping assays.

FIGURE S3 Tmod1 and Tmod2 overexpression does not alter soma area. Soma areas from 29–55 control neurons and Tmod1 or Tmod2 overexpressing neurons were analyzed at 12 DIV. No differences in the mean soma area were detected between groups (one-way ANOVA, *P* < 0.05).

FIGURE S4 Representative images of spine morphology affected by Tmod overexpression. **(A)** Spine morphology of in neurons at 12 DIV was visualized by expression of RFP-tagged actin. Scale bars 5 μm. **(B,C)** Spine categorization. **(B)** Explanation of criteria for spine categorization. Spines were manually counted as filopodia/thin spines, mushroom spines, or stubby spines. Protrusions were counted as filopodia/thin spines if the diameter of the neck (dn) was less than the length (L) and less than or equal to the diameter of the head (dh). Protrusions were counted as mushroom spines if the diameter of the neck was significantly less than the diameter of the head. Protrusions were counted as stubby spines if the diameter of the head was that of the diameter of the neck. **(C)** Example of spine counting: 1 indicates a thin spine, 2 indicates a mushroom spine, 3 indicates a stubby spine. Not every spine was marked in this image due to limited space.

FIGURE S5 Destroying the ABS1 or ABS2 does not alter soma area or the area of the neurite tips. Neurons, overexpressing non-mutant or mutant Tmod1 or Tmod2 were analyzed at 3 DIV. **(A)** Soma areas from 9–15 neurons and **(B)** neurite tips from 23–51 neurites were analyzed per condition. No differences in the mean soma area were detected between groups (one-way ANOVA for normally distributed or Kruskal-Wallis for non-normal distributed data, *P* < 0.05).
