# Supplementary figures and images for: Tropomodulin’s Actin-Binding Abilities Are Required to Modulate Dendrite Development
Source: Front Mol Neurosci. 2018 Oct 9;11:357. doi: 10.3389/fnmol.2018.00357 (PMC6190845; doi:10.3389/fnmol.2018.00357)

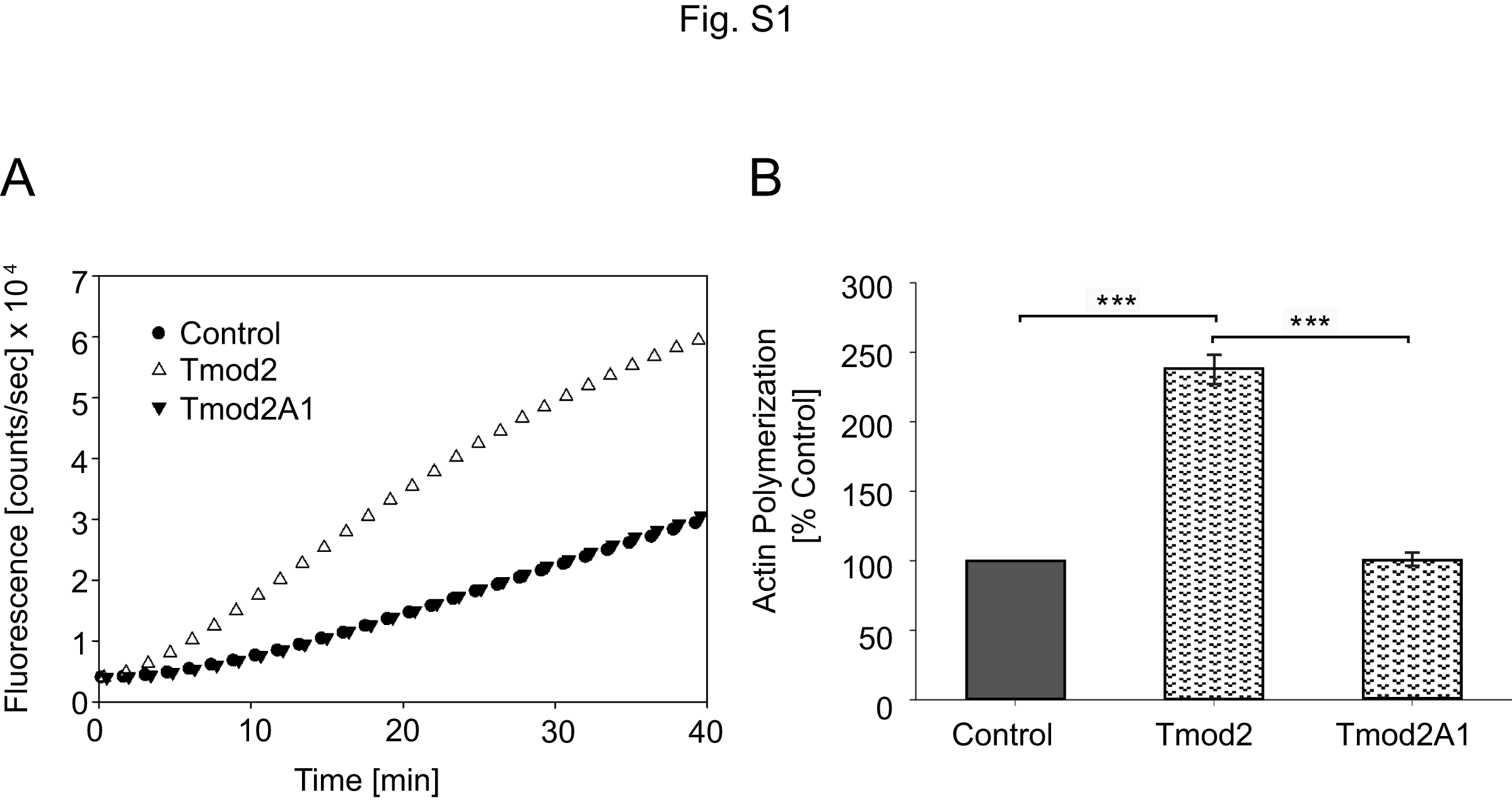

Supplement: FIGURE S1 — The L73D mutation abolishes Tmod2’s nucleation ability. (A) Actin nucleation by 200 nM Tmod2 or Tmod2A1, control is actin alone. (B) Degree of actin polymerization calculated as a percentage of corresponding fluorescence in the control. Averages and standard deviations (n = 4–5) are demonstrated by columns and error bars, respectively. Asterisks indicate statistically significant differences between groups (***p < 0.001) using one-way ANOVA with Holm-Sidak post hoc test. [file Image_1.TIF]

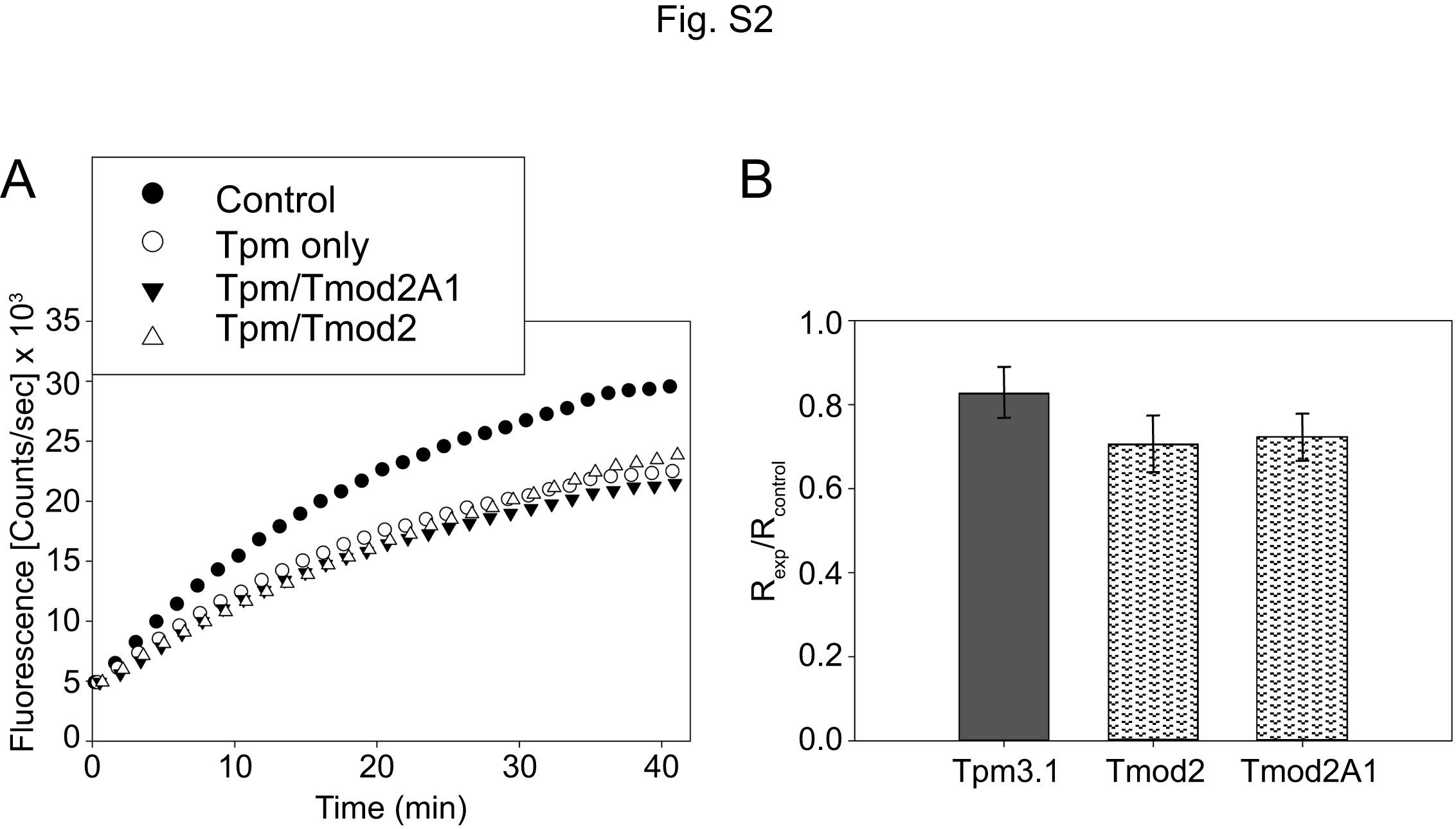

Supplement: FIGURE S2 — Capping activity of Tmod2 and mutant Tmod2A1. (A) Pyrene actin pointed end-capping assay. (B) Comparison of fluorescence measurements after 40 min for pyrene actin pointed end capping assays. [file Image_2.TIF]

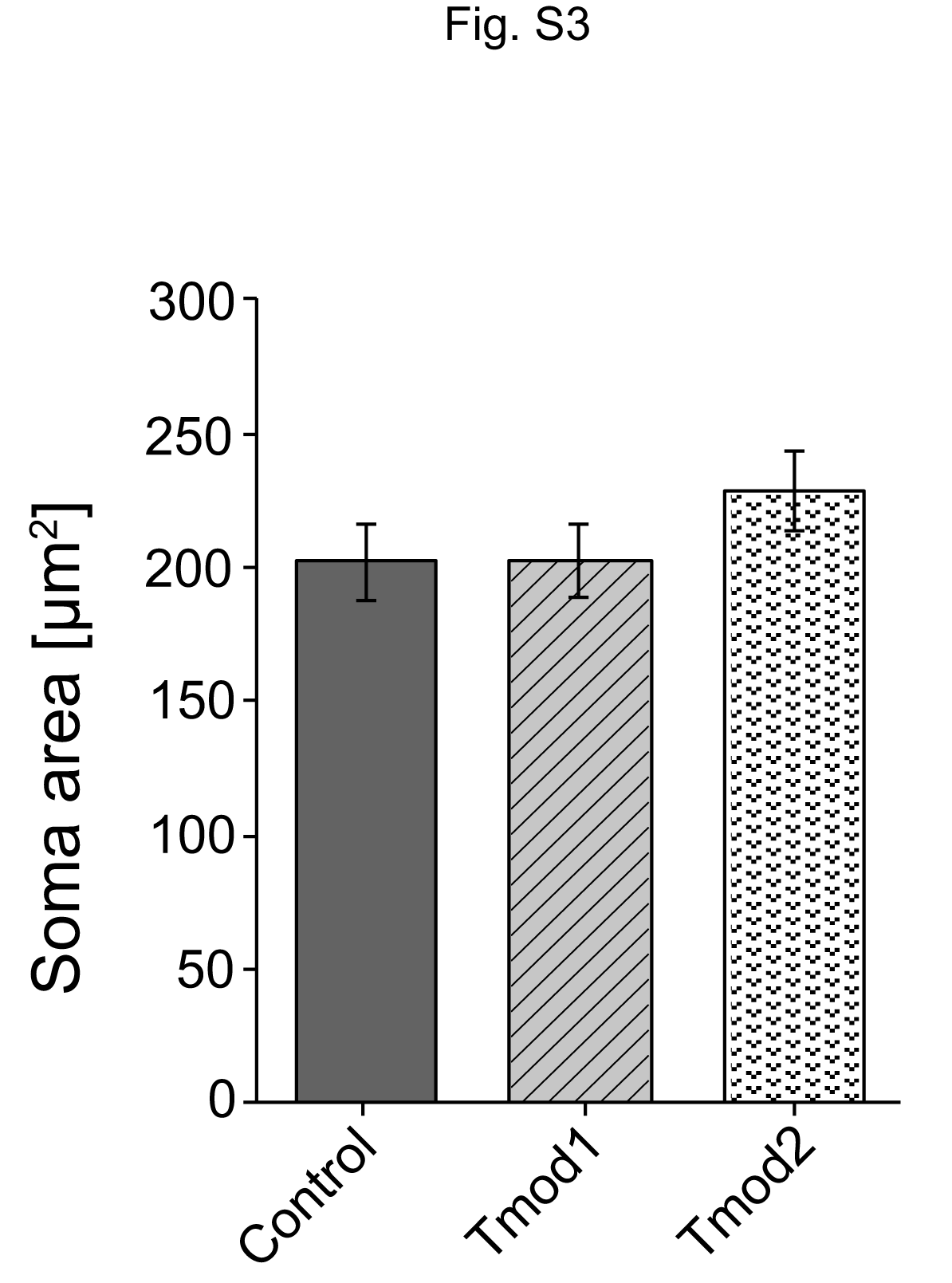

Supplement: FIGURE S3 — Tmod1 and Tmod2 overexpression does not alter soma area. Soma areas from 29–55 control neurons and Tmod1 or Tmod2 overexpressing neurons were analyzed at 12 DIV. No differences in the mean soma area were detected between groups (one-way ANOVA, P < 0.05). [file Image_3.TIF]

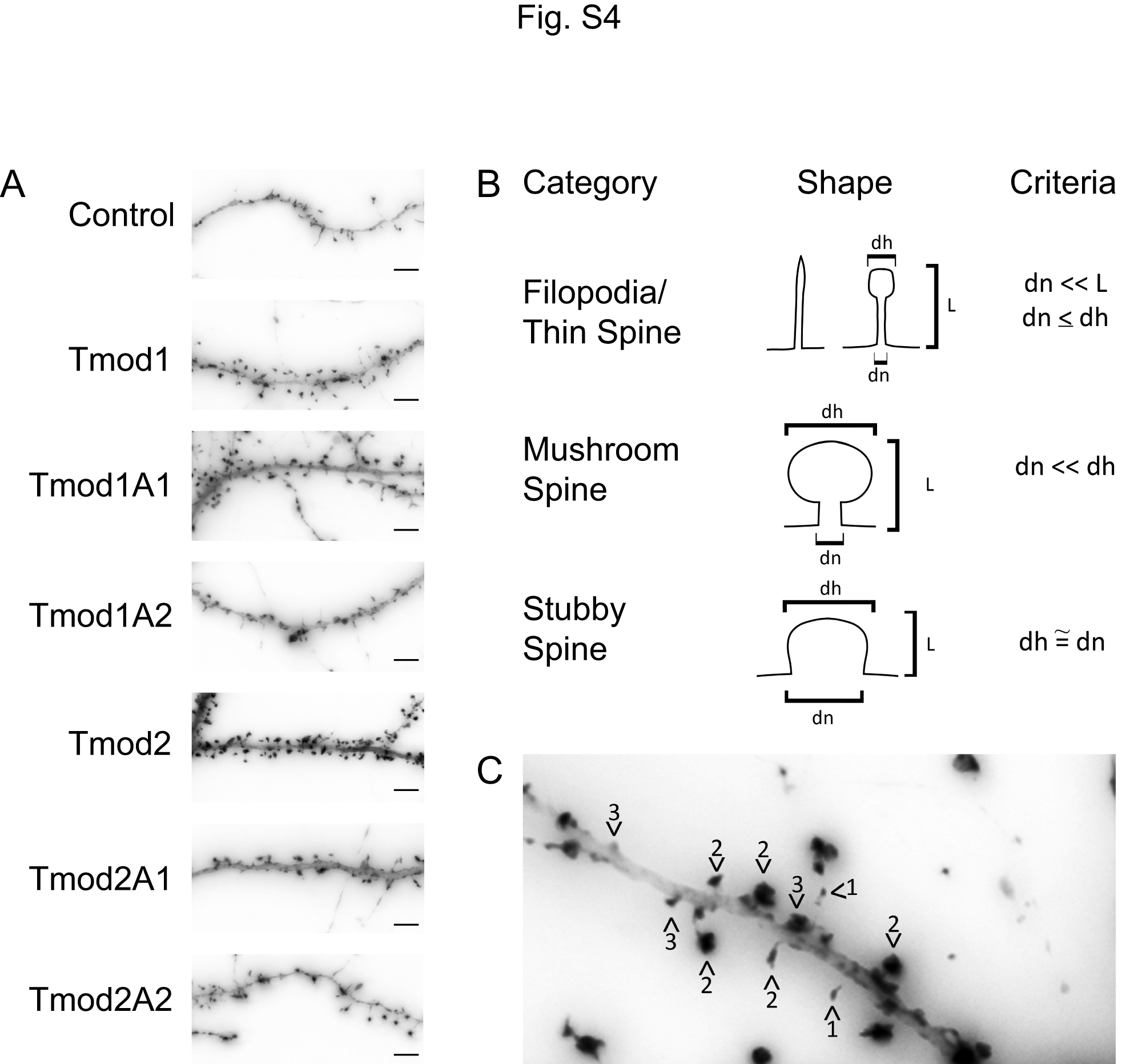

Supplement: FIGURE S4 — Representative images of spine morphology affected by Tmod overexpression. (A) Spine morphology of in neurons at 12 DIV was visualized by expression of RFP-tagged actin. Scale bars 5 μm. (B,C) Spine categorization. (B) Explanation of criteria for spine categorization. Spines were manually counted as filopodia/thin spines, mushroom spines, or stubby spines. Protrusions were counted as filopodia/thin spines if the diameter of the neck (dn) was less than the length (L) and less than or equal to the diameter of the head (dh). Protrusions were counted as mushroom spines if the diameter of the neck was significantly less than the diameter of the head. Protrusions were counted as stubby spines if the diameter of the head was that of the diameter of the neck. (C) Example of spine counting: 1 indicates a thin spine, 2 indicates a mushroom spine, 3 indicates a stubby spine. Not every spine was marked in this image due to limited space. [file Image_4.TIF]

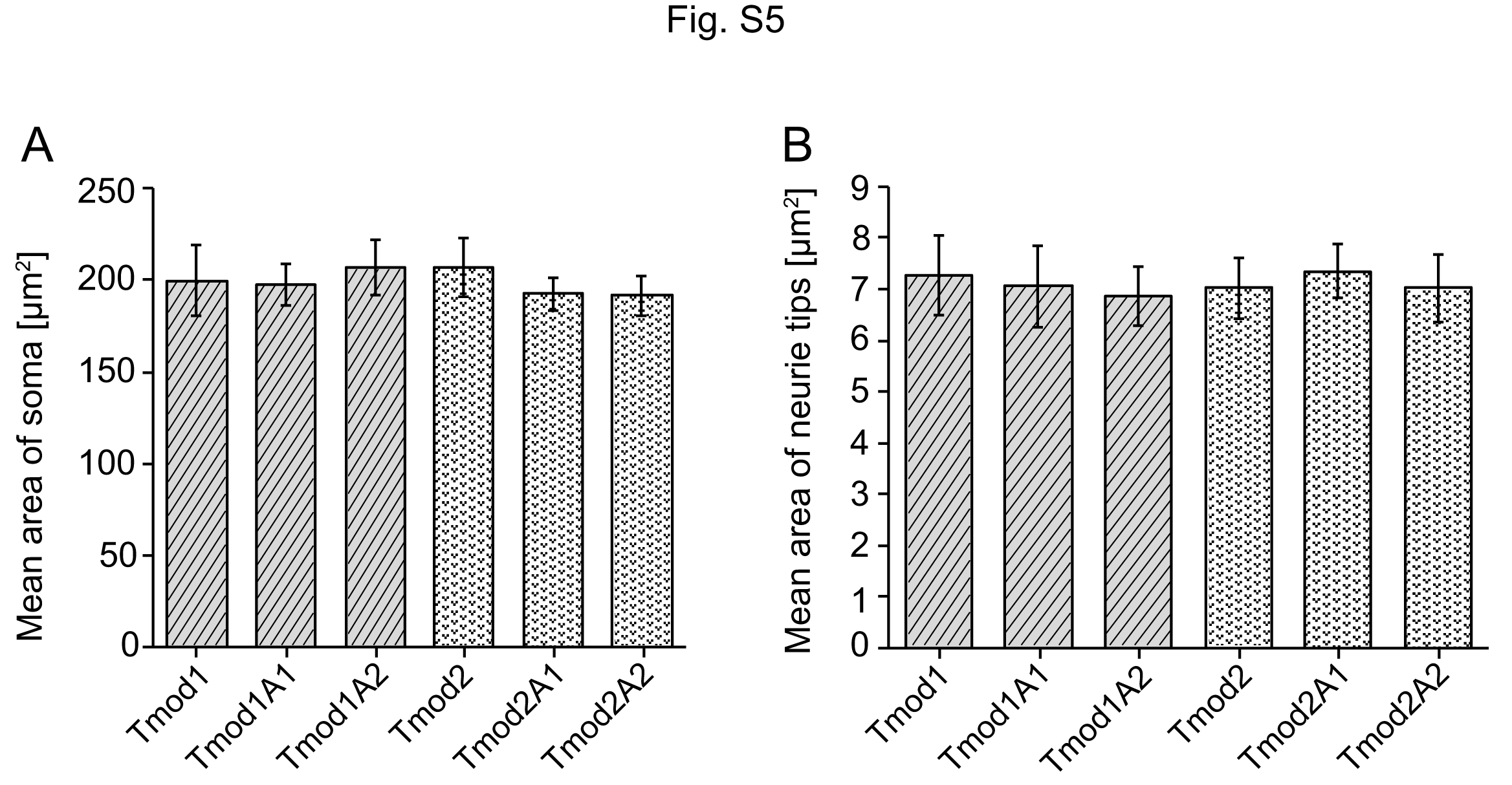

Supplement: FIGURE S5 — Destroying the ABS1 or ABS2 does not alter soma area or the area of the neurite tips. Neurons, overexpressing non-mutant or mutant Tmod1 or Tmod2 were analyzed at 3 DIV. (A) Soma areas from 9–15 neurons and (B) neurite tips from 23–51 neurites were analyzed per condition. No differences in the mean soma area were detected between groups (one-way ANOVA for normally distributed or Kruskal-Wallis for non-normal distributed data, P < 0.05). [file Image_5.TIF]
